# Inhibition of HIV replication *in vitro* by clinical immunosuppressants and chemotherapeutic agents

**DOI:** 10.1186/2045-3701-3-22

**Published:** 2013-05-14

**Authors:** Todd Hawley, Mark Spear, Jia Guo, Yuntao Wu

**Affiliations:** 1 National Center for Biodefense and Infectious Diseases, Department of Molecular and Microbiology, George Mason University, 10900, University Boulevard, Manassas, VA, USA

**Keywords:** HIV-1, Rev-CEM, CD4 T cells, Chemotherapy, Mycophenolic acid, Cyclosporine, Cytarabine

## Abstract

**Background:**

Recent studies have suggested that a functional cure for HIV-1 infection, purportedly resultant from allogeneic bone marrow transplantation, may be possible. Additionally, the first such patient was treated with whole-body irradiation, immunosuppressants, and the chemotherapeutic, cytarabine. However, the precise role of the coinciding medical interventions in diminishing detectable HIV reservoirs remains unstudied.

**Findings:**

In this article, we demonstrate that the immunosuppressants, mycophenolic acid and cyclosporine, and the chemotherapeutic, cytarabine, are potent antiretroviral agents at clinically relevant dosages. These drugs strongly inhibit HIV-1 replication in a GFP indicator T cell line and peripheral blood mononuclear cells (PBMC).

**Conclusions:**

Our study suggests that certain clinical immunosuppressants and chemotherapeutic agents may act combinatorially to inhibit HIV infection. Additionally, chemotherapy-mediated cytotoxicity may also affect the stability of viral reservoirs. Thus, further study is needed to examine potential therapeutic value of these interventions in patients.

## Findings

## Materials and methods

Supplementary Materials and Methods can be found in Additional file 1.

In 2009, a report described the first patient (the Berlin patient) to be possibly cured of HIV infection [1]. This HIV patient also presented acute myeloid leukemia, which was treated with allogeneic stem cell transplantation from a donor lacking functional HIV CCR5 coreceptor expression [1]. As of this publication, and four years after removal of Antiretroviral Therapy (ART), the patient exhibits undetectable plasma mRNA and viral reservoirs [2]. This unique case led to the speculation that it is possible to functionally cure HIV through mutagenesis of CCR5 [2,3].

More recently, two patients in Boston, as was announced at the 19th International AIDS Conference, have undergone similar bone marrow transplantation, with indications of possible diminishment of viral reservoirs [4]. However, the allogeneic hematopoietic stem cells given to the Boston patients were derived from donors that express functional CCR5 receptors. This raises the question in regards to what actually may have caused the decay of viral reservoirs in the Berlin patient. Common procedures among these patients include whole-body irradiation, chemotherapy, and immunosuppressant treatment, and it is possible that some of these procedures may have contributed to reduction of viral reservoirs [1,2]. Significantly, as the invasiveness of bone marrow transplantation renders it inapplicable for most HIV patients, illustration of the clinical benefits of these pharmacological interventions may lead to a novel methodology for HIV reservoir elimination.

**Figure 1:**
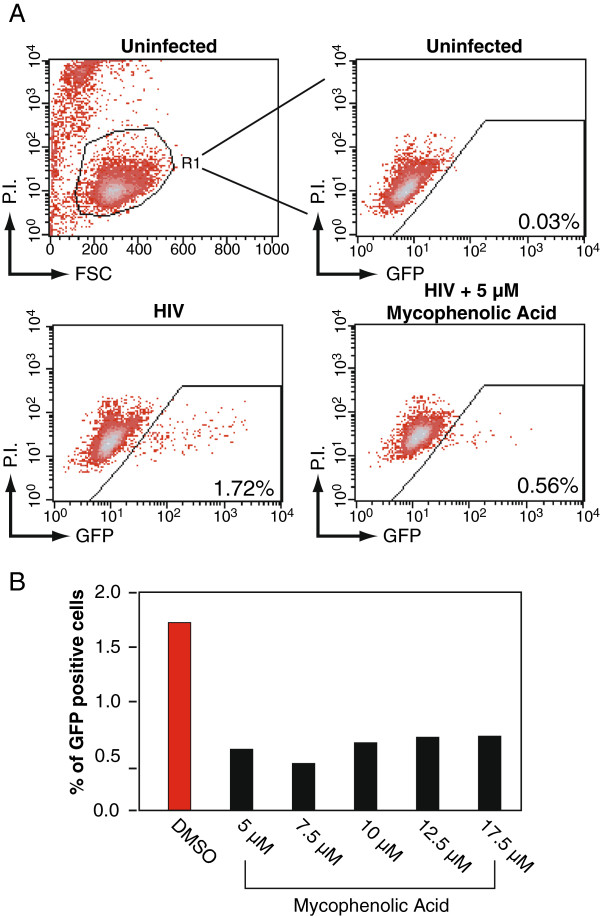
**MMF inhibits HIV-1 replication in Rev-CEM.** (**A**) Rev-CEM GFP indicator cells were treated with the indicated MMF concentration for 2 hours prior to infection with 200 ng HIV_NL4-3_ for two hours. Cells were washed and cultured for 48 hours, and analyzed with flow cytometry. To exclude cytotoxicity, cells were also stained with propidium iodide (P.I.) and GFP expression was measured only in the viable cell population. (**B**) The effect of MMF on HIV replication as a function of concentration at 48 hours post-infection.

In this article, we tested possible HIV-inhibitory effects of the chemotherapeutic agent and immunosuppressants given to the Berlin patient. The immunosuppressants mycophenolate mofetil (MMF) and cyclosporine were given to the patient to prevent rejection of the allogeneic stem cell transplant, whereas the chemotherapeutic agent cytarabine was utilized to kill leukemic cells. To test the effect of mycophenolic acid (MMF) on HIV-1 replication, a GFP indicator cell line, Rev-CEM, [5,6] was utilized. Briefly, cells were treated with MMF at the indicated dosages (Figure 1A), followed by infection with HIV_NL4-3_. At 48 hours post-infection, cells were analyzed for GFP expression, which measures the degree of HIV-1 replication. Infected cells were also stained for apoptosis with propidium iodide (P.I.) during flow cytometry to exclude drug cytotoxicity so that GFP expression will be measured only in the viable cell population. We observed that MMF inhibited HIV-1 replication at all tested dosages (Figure 1A and 1B) (48 hours post-infection), in agreement with a previous report [7,8]. Notably, MMF inhibited HIV-1 replication at clinically relevant dosages (1–10 μM) [9].

**Figure 2:**
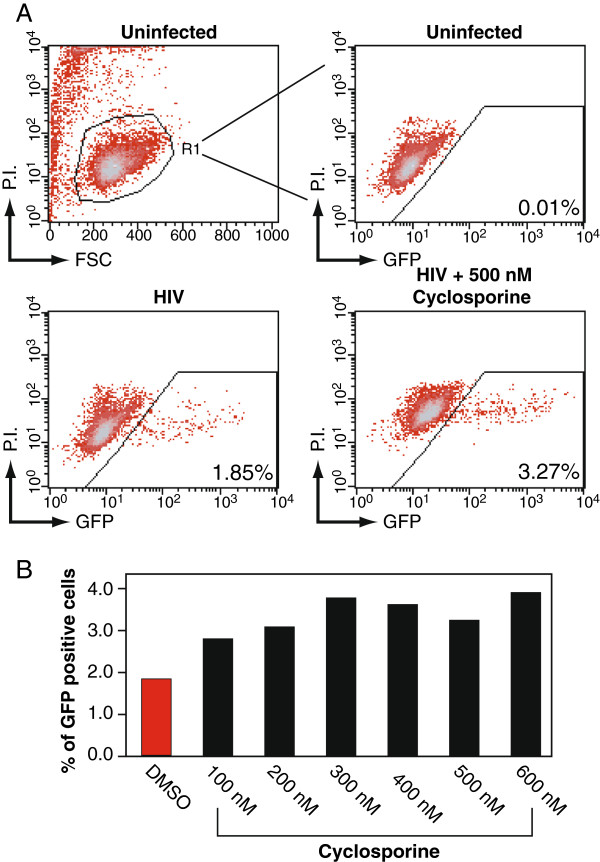
**Cyclosporine does not suppress HIV-1 replication in Rev-CEM.** (**A**) Rev-CEM GFP indicator cells were treated with the indicated cyclosporine concentration for 2 hours prior to infection with 200 ng HIV_NL4-3_ for two hours. Cells were washed and cultured for 48 hours, and similarly analyzed with flow cytometry as described in Figure 1A. (**B**) The effect of cyclosporine on HIV replication as a function of concentration at 48 hours post-infection.

Similarly, cyclosporine-mediated HIV-1 inhibition was measured as above (Figure 2A). However, no such inhibition was observed at any of the indicated dosages (Figure 2B). To the contrary, slight enhancement was observed in all the dosages tested. As the effects of cyclosporine A on HIV replication are markedly dependent on the cell type, with cell lines typically showing modest effects, it is quite possible that inhibition may not be observable utilizing this HIV Rev-dependent indicator cell line [10].

**Figure 3:**
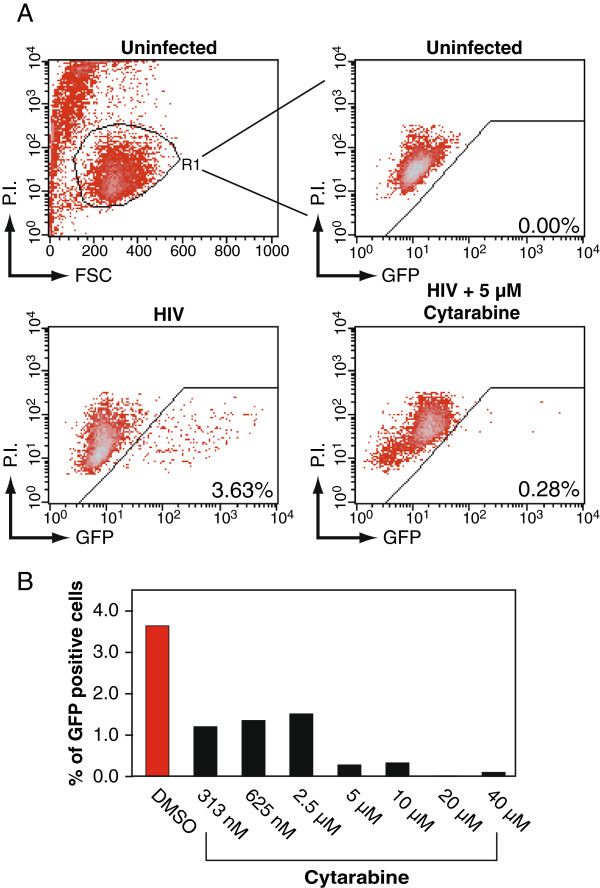
**Cytarabine inhibits HIV-1 replication in Rev-CEM.** (**A**) Rev-CEM GFP indicator cells were treated with the indicated cytarabine concentration for 2 hours prior to infection with 200 ng HIV_NL4-3_ for two hours. Cells were washed and cultured for 48 hours, and similarly analyzed with flow cytometry as described in Figure 1A. (**B**) The effect of cytarabine on HIV replication as a function of concentration at 48 hours post-infection.

Cytarabine (Ara-C), the major chemotherapeutic agent administered to the Berlin patient, was also assayed for antiretroviral activity as above (Figure 3A). At all indicated dosages, cytarabine inhibited HIV replication (Figure 3B). Importantly, Ara-C inhibited HIV at the therapeutic doses (200 nM – 50 µM) [11]. More significant inhibition was observed at higher dosages (Figure 3B).

**Figure 4:**
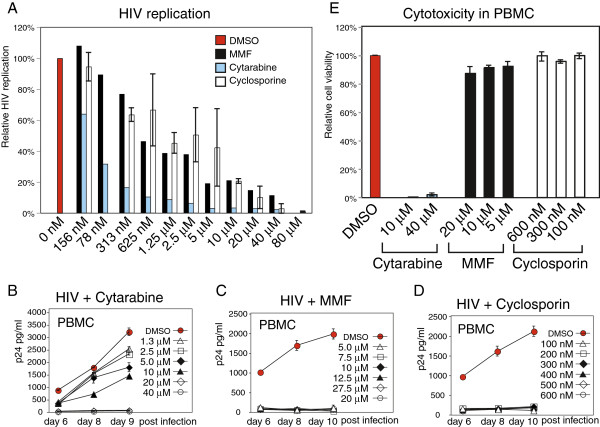
**Cytarabine, mycophenolic acid, and cyclosporine inhibit HIV-1 replication in Rev-CEM-Luc and PBMC.** (**A**) Rev-CEM-Luc indicator cells were treated with the indicated drugs for 2 hours prior to infection with HIV_NL4-3_ for two hours. Cells were washed and cultured for 48 hours, and analyzed for HIV-dependent luciferase activity. (**B** to **D**) PMBCs were purified from healthy donors. For HIV infection, cells were pre-activated with PHA plus IL-2 for 24 hours. One million cells were treated with each of the reagents for 2 hours, and then infected with HIV-1 (250 ng p24) for 2 hours in the presence of the reagents. Infected cells were washed and resuspended in 1 ml of fresh medium with PHA plus IL-2, and the reagents added. Additional PHA plus IL-2 were added every 48 hours. Supernatant was taken for measuring viral p24 release by ELISA. (**B**) Cytarabine dosage-dependent inhibition of HIV-1 infection of PBMC. (**C**) MMF dosage-dependent inhibition of HIV infection of PBMC. (**D**) Cyclosporine dosage-dependent inhibition of HIV infection of PBMC. (**E**) To measure drug cytotoxicity, PMBC were also stained with propidium iodide (P.I.) and measured with flow cytometry. Shown are results at day 10 post drug treatment.

To confirm these findings, we used a complementary HIV Rev-dependent reporter cell, Rev-CEM-Luc, which expresses luciferase following HIV infection. We observed drug dosage-dependent inhibition of HIV-mediated luciferase activity (Figure 4A). The Rev-CEM-Luc cell is more quantitative in measuring inhibition of HIV, because the luciferase activity reflects the overall viral activity. On the other hand, Rev-CEM measures GFP cell percentage which only reflects the relative numbers of cells infected. These two reporter systems complement each other, as Rev-CEM-Luc, although more quantitative, cannot easily measure the number of HIV+ cells, and possible drug cytotoxicity.

To further confirm these results in primary blood cells, we isolated PBMCs from healthy donors and treated cells with inhibitors to determine inhibition of HIV replication. For HIV infection of PBMC, cells were pre-activated with PHA plus IL-2 for 24 hours. One million cells were treated with each of the reagents for 2 hours, and then infected with HIV-1 for 2 hours in the presence of the reagents. Infected cells were washed and resuspended in 1 ml of fresh medium with PHA plus IL-2, and the reagents added. Inhibition of HIV replication was measured by the decrease of HIV p24 release in the supernatant. As shown in Figure 4B, Cytarabine exhibited inhibition of HIV at all dosages tested. Particularly, it completely inhibited HIV at dosages above 10 μM, dosages that are clinically achievable during chemotherapeutic administration [11]. Mycopheonolic Mofetil exhibited similar inhibition at all indicated dosages (Figure 4C), largely recapitulating previously reported data [7]. Similarly, exquisite inhibition of HIV-1 was attained with cyclosporine at all concentrations tested, including those that were subtherapeutic [12]. We also measured the cytotoxicities of these drugs in PMBC. Cells were identically activated and treated with these drugs, and apoptotic cells were measured by propidium iodide staining. As shown in Figure 4E, high dosages of the chemotherapeutic drug cytarabine showed cytotoxicity in PMBC, whereas the other two drugs have no detectable cytotoxicity at all the dosages tested. The inhibition of HIV replication by cytarabine likely resulted from direct killing of HIV-infected PBMC.

The diminishment of viral rebound exhibited by the Berlin patient, as is currently indicated, likely resulted from the removal of persistent viral reservoirs by some unknown combination of factors; these may include chemotherapy (cytarabine), immunosuppression (antithymocyte globulin, cyclosporine, and MMF), whole-body irradiation, allogeneic stem cell transplantation, and some degree of graft-versus-HIV immunoreactivity [2]. In this paper, we report the potent antiretroviral activity of cytarabine in an indicator T cell line, REV-CEM. Cytarabine also diminished HIV-infected PBMC likely by direct killing of infected cells. Furthermore, we recapitulate findings of the antiretroviral activity of cyclosporine and MMF in PBMC culture. Together, our results indicate that there is a possibility that a combined use of irradiation, drug-mediated immunosuppression, ART, and chemotherapy-induced cytotoxicity may lead to viral suppression and synergistically increased the decay of viral reservoirs. The therapeutic potential of these treatments, in the absence of transplantation, on viral reservoirs deserves further clinical investigation.

## Competing interests

The authors declare that they have no competing interests.

## Authors’ contributions

TH, MS, JG performed the experiments and analyzed the results. YW conceived the study. MS and YW prepared the manuscript. All authors read and approved the final manuscript.

## Supplementary Material

Additional file 1 Supplementary Materials and Methods. Click here for file
