# Supplementary material for: Inhibition of HIV replication in vitro by clinical immunosuppressants and chemotherapeutic agents
Source: Cell Biosci. 2013 May 14;3:22. doi: 10.1186/2045-3701-3-22 (PMC3680316; doi:10.1186/2045-3701-3-22)
Supplement: Additional file 1 — Supplementary Materials and Methods. [file 2045-3701-3-22-S1.doc]

Supplementary Materials and Methods

**Inhibition of HIV replication *in vitro* by clinical immunosuppressants and chemotherapeutic agents**

Todd Hawley, Mark Spear, Jia Guo, and Yuntao Wu*

National Center for Biodefense and Infectious Diseases

Department of Molecular and Microbiology

George Mason University

10900 University Boulevard

Manassas, VA, USA

* To whom correspondence should be addressed. Email: ywu8@gmu.edu

**This PDF file includes:**

Materials and Methods

References

**Materials and Methods**

**Cell and Viruses:** All protocols involving human subjects were reviewed and approved by the George Mason University (GMU) institutional review board. Peripheral mononuclear cells (PBMC) were purified from the peripheral blood of healthy donors from GMU using lymphocyte separation medium (Mediatech) as previously described [1]. Purified cells were cultured in RPMI 1640 medium supplemented with 10% heat-inactivated fetal bovine serum (Invitrogen), penicillin (50 U/ml) (Invitrogen), streptomycin (50 mg/ml) (Invitrogen), and phytohaemagglutinin (PHA) (3 µg/ml) (Sigma) plus IL-2 (100 U/ml) (Roche Applied Science). The HIV Rev-dependent GFP indicator T cell lines, Rev-CEM and Rev-CEM-Luc, have been previously described [2, 3], and was cultured in RPMI 1640 complete medium. Virus stocks of HIV-1_NL4-3_ [4] were prepared by transfection of HEK393T cells with cloned proviral DNA as described [5]. Levels of p24 in the viral supernatant were measured in triplicate on the same ELISA plates using the PerkinElmer Alliance p24 antigen ELISA Kit (PerkinElmer). Viral titer (TCID_50_) was determined on Rev-CEM [2, 3].

**Drug Treatment and HIV Infection**: Cytarabine, mycophenolic acid, and cyclosporin were acquired from the NIH Developmental Therapeutics Program. These reagents were dissolved in dimethyl sulfoxide (DMSO) (Sigma). For infection of Rev-CEM or Rev-CEM-Luc, 2 x 10^5^ cells were pretreated with each of the above reagents for one hour at 37ºC. Cells were centrifuged at 300 x g for 5 minutes to remove the supernatant, and then infected with 100 μl of HIV-1 (250 ng p24) in the presence of the reagents. Cells were infected for two hours at 37ºC, washed, and then re-suspended into 1 ml medium with the drugs added. Infected cells were cultured for two days and analyzed for GFP expression or luciferase activity with flow cytometry on FACSCalibur (BD Biosciences) or GloMax Multi Detection system (Promega). Propidium iodide (Fluka) was added (2 µg/ml) before flow cytometry to exclude dead cells. For HIV infection of PBMC, cells were pre-activated with PHA plus IL-2 for 24 hours. One million cells were treated with each of the reagents for 2 hours, and then infected with HIV-1 (250 ng p24) for 2 hours in the presence of the reagents. Infected cells were washed and resuspended in 1 ml of fresh medium with PHA plus IL-2, and the reagents added. Additional PHA plus IL-2 were added every 48 hours. Drug cytotoxicity was monitored by staining PBMC with Propidum iodide and flow cytometry as described above. Levels of p24 in the supernatant were measured in triplicate on ELISA plates using the PerkinElmer Alliance p24 antigen ELISA Kit (PerkinElmer) or an in-house p24 ELISA kit. Briefly, each well of a plate was coated with capture antibody, incubated overnight at room temperature, and then washed and blocked with blocking solution for 1 hour at room temperature. Samples in plates were incubated for 1 hour at 37ºC, and then washed and incubated with biotin-labelled detection antibody for 1 hour at 37ºC. Plates were washed and incubated with avidin-peroxidase conjugate for 30 minutes at room temperature followed by washing and incubation with tetramethylbenzidine (TMB) substrate buffer. Plates were kinetically read using an ELx808 automatic microplate reader (Bio-Tek Instruments) at 630 nm.

**References:**

1. Yoder A, Yu D, Dong L, Iyer SR, Xu X, Kelly J, Liu J, Wang W, Vorster PJ, Agulto L, et al: HIV envelope-CXCR4 signaling activates cofilin to overcome cortical actin restriction in resting CD4 T cells. *Cell* 2008, 134:782-792.

2. Wu Y, Beddall MH, Marsh JW: Rev-dependent lentiviral expression vector. *Retrovirology* 2007, 4:12.

3. Wu Y, Beddall MH, Marsh JW: Rev-dependent indicator T cell line. *Current HIV Research* 2007, 5:395-403.

4. Adachi A, Gendelman HE, Koenig S, Folks T, Willey R, Rabson A, Martin MA: Production of acquired immunodeficiency syndrome-associated retrovirus in human and nonhuman cells transfected with an infectious molecular clone. *J Virol* 1986, 59:284-291.

5. Wu Y, Marsh JW: Selective transcription and modulation of resting T cell activity by preintegrated HIV DNA. *Science* 2001, 293:1503-1506.
